# Interventions to Improve Mealtime Care Quality and Outcomes in Persons Living with Dementia and Their Caregivers: A Systematic Review and Meta‐Analysis

**DOI:** 10.1002/alz.088427

**Published:** 2025-01-09

**Authors:** Wen Liu, Heather Suh, Kyuri Lee

**Affiliations:** ^1^ The University Of Iowa, Iowa City, IA USA

## Abstract

**Background:**

Mealtime is a fundamental daily activity to ensure nutrition, social interaction, and enjoyment of food for people with dementia (PWD). Interventions addressing multilevel barriers are critical to optimize mealtime care and outcomes. This review aimed to synthesize existing interventions on mealtime care and outcomes in PWD and their caregivers.

**Method:**

PubMed, CINAHL, AgeLine, PsycINFO, and Cochrane were searched from January 2012 to May 2023 using keywords including dementia, Alzheimer, food intake, and mealtime. Relevant bibliographies were manually searched. Eligibility criteria were developed by defining the population, intervention, comparator, outcome, timing, and setting of interest. Eligible studies were classified by intervention, accessed for quality using the Quality Assessment Tool for Quantitative Studies, and graded for evidence using the Grading of Recommendations, Assessment, Development and Evaluation Working Group criteria. Meta‐analyses were performed for studies within the same intervention type that tested the impact on same outcomes.

**Result:**

Of 1,137 records, 30 studies were identified and categorized into five intervention types: nutritional supplements (5), resident training/therapy (7), Dyadic training or mealtime interaction (7), environmental/food modification (7), and multiple‐component interventions (4). “Nutritional supplements” showed low evidence on cognition and nutritional status, and moderate evidence on ADL function and depression. “Resident training/therapy” showed moderate evidence on cognitive function. All intervention types showed very low to low evidence on food intake, weight, and BMI, eating difficulties, and hyperphagic behaviors. Meta‐analyses of limited number of heterogenous studies showed 1) “Environmental/food modification” increased food intake; 2) “Resident training/therapy” decreased eating difficulties, 3) “Resident training/therapy” did not increase food intake, 4) “Dyadic training or mealtime interaction” did not increase BMI, 5) “Nutritional supplements” did not improve cognition, and 6) “Multiple components” did not improve ADL function.

**Conclusion:**

Findings support evidence of certain interventions on food intake and mealtime behaviors, and provide directions for clinical practice and research. Current evidence is based on a body of research with primarily moderate to weak quality. Future testing of interventions using high‐quality designs is needed.

